# Frequency and Characterisation of Anomalies and Fractures of the Caudal Spine in Sheep with Undocked Tails

**DOI:** 10.3390/ani13081419

**Published:** 2023-04-21

**Authors:** Hannah Hümmelchen, Henrik Wagner, Kerstin Brügemann, Sabine Wenisch, Sven König, Axel Wehrend

**Affiliations:** 1 Clinic for Obstetrics, Gynaecology and Andrology of Small and Large Animals, Justus-Liebig University of Giessen, 35392 Giessen, Germany; 2 Institute for Animal Breeding and Domestic Animal Genetics, Justus-Liebig University of Giessen, 35398 Giessen, Germany; 3 Institute of Veterinary Anatomy, Histology and Embryology, Justus Liebig University of Giessen, 35392 Giessen, Germany

**Keywords:** X-ray examination, malformation, block vertebra, wedged vertebra, vertebral fractures

## Abstract

**Simple Summary:**

Due to the widespread practice of tail docking in sheep, no data exist regarding the incidence of caudal spine deformities and injuries in this species. The aim of this study was to describe the type and frequency of tail abnormalities in an undocked Merinoland sheep population using radiographic studies. The findings demonstrated that anomalies of the tail bones (such as fractures and block or wedged vertebrae) were predominantly found in the middle and caudal third of the spine. Additionally, a correlation between the occurrence of vertebral fractures and the tail length and number of vertebrae was observed. No significant differences were found in terms of different litter sizes and sex. These results suggest that the proportion of animals with tail malformations and injuries is relatively high, which should be considered when breeding for short tails in the context of a docking ban.

**Abstract:**

As tails are often docked within the first days of life, studies investigating tail malformations and injuries in sheep do not exist thus far. To address this gap in the literature, this research aimed to analyse the occurrence of vertebral anomalies and fractures in the tail within an undocked Merinoland sheep population. At 14 weeks of age, the caudal spines of 216 undocked Merinoland lambs was radiographically examined, and tail length and circumference were measured. Anomalies were documented and statistical correlation and model calculations were performed. The occurrence of block vertebrae was observed in 12.96% and wedged vertebrae in 8.33% of the sample. Of the animals, 59 (27.31%) exhibited at least one vertebral fracture, which were observed in the middle and caudal third of the tail. A significant correlation was found between the occurrence of fractures and tail length (r = 0.168) and number of vertebrae (r = 0.155). Conversely, the presence of block and wedged vertebrae was not significantly correlated with tail length, circumference, or number of vertebrae. Only the sex showed significant differences in the probability of axis deviation. These results emphasize the importance of breeding for short tails to avoid fractures.

## 1. Introduction

Despite legal prohibitions across countries, many sheep have their tails docked after birth [1]. At the European level, painful interventions on animals, such as tail docking in sheep, are not clearly regulated. In addition to EU directives, which provide the basis for the respective national regulations, there are also recommendations from the Council of Europe as well as EU regulations for organically managed farms. In the Council of Europe Recommendation for Sheep Husbandry, article 30 paragraph 2 allows tail docking in sheep as an exception to article 30 paragraph 1 [2]. Article 18 “Handling of animals” paragraph 1 of the Implementing Regulation of the EU Organic Regulation (EC) No 889/2008 prohibits the routine application of rubber rings to the tails of sheep. Nevertheless, the competent authority may authorize this intervention for safety reasons or to improve the health, welfare, or hygiene conditions of the animals. However, any suffering of the animals must be minimized by the use of appropriate anaesthetics and/or analgesics [3].

At the national level, there are major differences regarding a ban on tail docking or different conditions under which tail amputation may be performed in sheep [1].

Originally, the ancestors of today’s domestic sheep possessed short tails. This is evidenced by the mouflon (the wild form of sheep), which has a short, thin tail [4]. Over the centuries, in the course of domestication, sheep were bred to have longer tails, primarily for economic advantages in wool production owing to increased body surface area and, thus, greater wool yield [4]. This led to the large tail length variance between different sheep breeds. Based on tail type, sheep can be divided into five main groups. Thin-tailed sheep include, for example, Merino and Romney with tails up to the hock or longer. Another group is represented by sheep with fleshy long tails to the fetlocks or longer from Sudan and Eritrea. Nordic short-tailed breeds such as Finn sheep and Romanov are counted among the short-tailed sheep, which also include Texel. Furthermore, two groups of fat-tailed sheep can be divided. These include Awassi, Karakul, and both African and Asian sheep. In Europe, the thin-tailed and short-tailed sheep are of particular importance [5]. In Germany, the Merinoland sheep, with a share of 30%, represents the most common breed of sheep.

As wool production and processing have become considerably less important in recent decades, the negative consequences of longer sheep tails have become evident. Reasons cited for partial amputation include the avoidance of soiling in the anogenital region, which can cause problems during mating, lambing, slaughter, and shearing [1,6]. Furthermore, an increased risk of fly maggot infestation with faeces in the anogenital area has been noted [6,7]. Female sheep are also at increased risk of wound infection with clostridia, which may cause serious disease patterns, such as tetanus, and vaginal and uterine blight [8].

In Australia, the practice of mulesing is carried out in addition to tail amputation to prevent fly maggot infestation [9]. During this procedure, skin folds in the anogenital area of merino sheep are removed without anaesthesia [10]. However, another Australian study showed that the extreme shortening of tails, even in combination with mulesing, again favours the contamination with faeces and, consequently, the occurrence of myiasis [11]. Further disadvantages of the practice of tail docking are known. Firstly, amputation inflicts pain on the animals, which produces pain-associated changes in behaviour and posture [12,13]. An additional study examined 63,287 sheep carcasses in South Australia and found a direct correlation between shortened tails (less than three caudal vertebrae) and the incidence of bacterial arthritides. The major pathogens detected from the altered joints were *Erysipelothrix rhusiopathiae* and *Streptococcus* spp. [14].

Finally, the incidence of potential naturally occurring tail malformations in the population cannot be recorded. As such, there is a risk that these abnormalities, if hereditary, could continue to spread through breeding. Therefore, the aim of this study was to investigate and morphologically describe the occurrence of tail anomalies and fractures in a Merinoland sheep population.

## 2. Materials and Methods

### 2.1. Animals and Data Collection

At the age of 14 weeks, 216 undocked Merinoland sheep from the research farm Oberer Hardthof of the Justus Liebig University were examined for the study. Until the age of four days, the lambs were kept in individual pens together with the corresponding Merino ewes and later on in small groups. From the second week of life, the lambs were fed self-produced hay and commercial concentrates. At 10 weeks of age, the lambs were weaned from their dams.

This examination included a radiographic examination of the tail bones and measurement of the length and circumference of the tails. Tail length and circumference, as well as the occurrence of vertebral malformations, fractures, and axial deviations in the tail were recorded. The examinations were approved by the Regional Council of Giessen (V 54—19c 20 15 h 01 GI 18/14 No. G 44/2021).

Metric measurements were carried out using a self-made construction with an integrated measuring tape and X-ray images were obtained with a portable X-ray device (Physia GAMMA Light AD 100|20). For the measurement of the tail, the lamb was held in a standing position and the tail was placed on the construction. Using a slide, the length of the tail could be accurately measured from the anus to the tip of the tail. The measurement of the tail circumference was performed with the help of an adjustable measuring tape attached to the same construction, through which the tail was placed. The tail circumference was measured at the most cranial region, just before the transition to the pelvis. For radiographic examination, the lambs were placed in a recumbent position and held fixed by an assistant. A setting of 40 kV and 2.5 mAs was used to image the vertebrae. Detailed information regarding the measurement methods can be found in Hümmelchen et al. [15].

**Figure 1:**
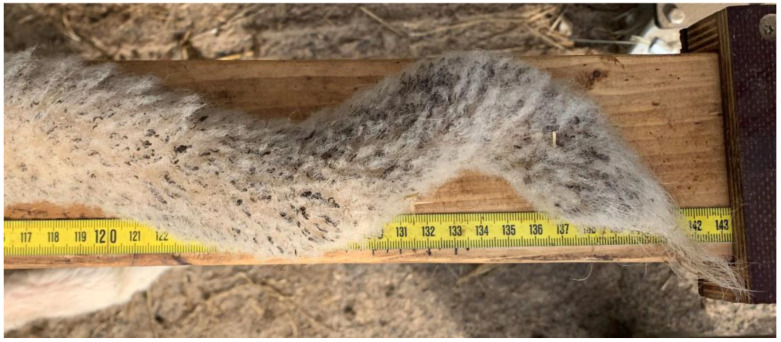
Sheep tail with axial deviation.

**Figure 2:**
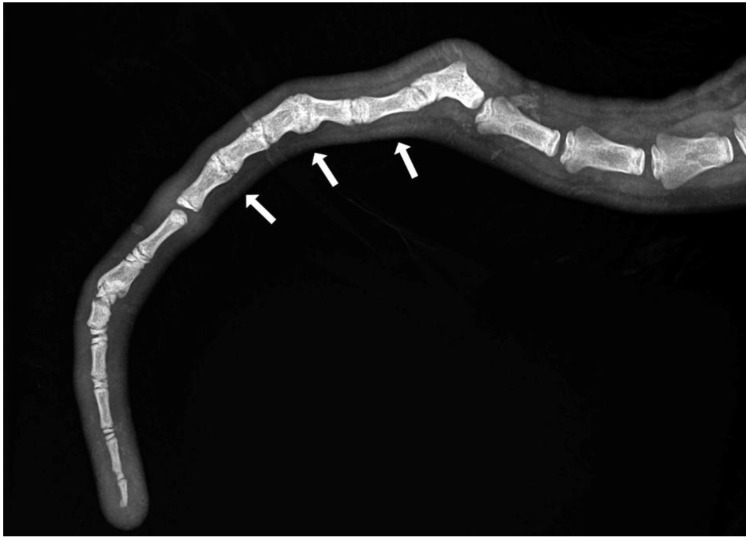
Section of an X-ray of a lamb’s tail with multiple block vertebrae.

**Figure 3:**
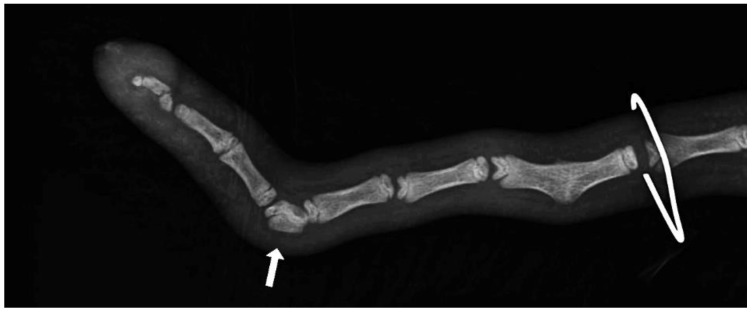
Section of an X-ray of a lamb’s tail with a wedged vertebra.

**Figure 4:**
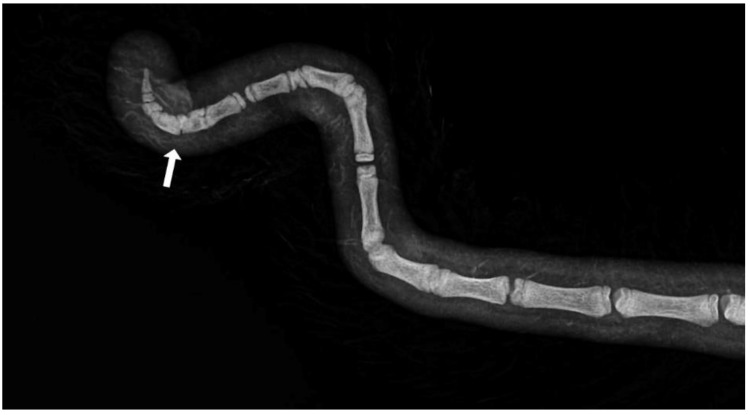
Section of an X-ray of a lamb’s tail with fractured vertebrae.

Axial deviation was defined as any manually irreparable deviation of the tail from the medians (Figure 1). A block vertebra was considered present if the vertebral boundaries of adjacent vertebrae were not clearly visible or were completely fused (Figure 2). Wedge-shaped bony structures present between two vertebrae were recorded as wedged vertebrae (Figure 3). A fracture was noted in any event where a vertebra demonstrated an interruption of bone structure (Figure 4).

### 2.2. Statistical Analyses

Phenotypic correlations were calculated to investigate the relationships between lamb traits such as tail length (‘TL’), tail circumference (‘TC’), number of vertebrae (‘nvertebrae’), and the incidence of vertebral lesions. The effect of litter size, sex, the number of times the dam has given birth, the ram, and the tail circumference on the incidence of tail malformations such as wedged vertebrae, block vertebrae, axial deviations, and fractures (binarily coded) was investigated using the following generalized linear models:

logit (π) = log [π_ijklmn_/(1 − π_ijklmn_)] = μ + litter size_i_ + sex_j_ + parity_k_ + ram_l_ + TCclass_m_ + nVclass_n_

where π_ijklmn_ = probability of occurrence for wedged vertebrae,

logit (π) = log [π_ijkln_/(1 − π_ijkln_)] = μ + litter size_i_ + sex_j_ + parity_k_ + ram_l_ + nVclass_n_

where π_ijkln_ = probability of occurrence for block vertebrae,

logit (π) = log [π_ijklo_/(1 − π_ijklo_)] = μ + litter size_i_ + sex_j_ + parity_k_ + ram_l_ + TLclass_o_

where π_ijklo_ = probability of occurrence for fractures,

logit (π) = log [π_ijklm_/(1 − π_ijklm_)] = μ + litter size_i_ + sex_j_ + parity_k_ + ram_l_ + TCclass_m_

where π_ijklm_ = probability of occurrence for axial deviation,

μ = the overall mean effect; litter size_i_ = fixed effect of ith litter size (i = single, twin, triplet); sex_j_ = fixed effect of jth sex (j = male, female); parity_k_ = fixed effect of kth parity (k = 1, 2, 3, >3); ram_l_ = fixed effect of lth ram (l = 1, 2, 3, 4); TCclass_m_ = fixed effect of mth TCclass (m = <13 cm, 13–14 cm, >14 cm); nVclass_n_ = fixed effect of nth nVclass (n =< 19, 19–20, >20), TLclass_o_ = fixed effect of oth TLclass (o =< 38 cm, 38–42 cm, >42 cm).

The model was analysed using the GLIMMIX procedure and corresponding LSMeans were calculated using a logit link function. The statistical programme SAS OnDemand for Academics (SAS, 2022) was used for this purpose. Further influencing factors such as the measuring age (deviation of up to five days) were not considered in the model due to the lack of significance. Only significant tail parameters or tail parameters that improved the model quality were included in the models for the respective characteristic. For this purpose, the effects were selected manually as well as using the variable selection method “forward selection” (procedure GLMSELECT).

## 3. Results

### 3.1. Measurements and Radiographic Examination

The 216 lambs comprised 106 (49%) males and 110 (51%) females. Within this group, 31 singles, 82 sets of twins, and 7 sets of triplets were born.

The mean number of tail vertebrae in the Merinoland sheep population was 20.4 (±1.6) vertebrae. The mean tail length at 14 weeks of life was 41.6 cm (±4.3 cm) and mean tail circumference measured at the same time was 13.15 cm (±1.3 cm). In 33 animals (15.28%), an axial deviation of the tail spine was detected. Radiographic examination revealed various spinal lesions, such as fractures (Figure 4), wedged vertebrae (Figure 3), and block vertebrae (Figure 2). In the caudal spine, at least one block vertebra was found in 28 animals (12.96%) and at least one wedged vertebra was detected in 18 animals (8.33%). A total of 59 animals (27.51%) exhibited at least one vertebral fracture within the caudal spine. In 51 of these 59 animals (86.4%), the fracture localisation was limited to the caudal third of the tail. Six animals (10.2%) had fractures in the middle third of the tail and two animals (3.4%) had vertebral fractures in both the middle and caudal third of the tail (Table 1).

Block vertebrae manifested primarily in the caudal and/or middle third of the tail (Table 2) and wedged vertebrae were exclusively in the caudal third of the tail (Table 3). Axial deviations in turn showed up in the caudal and/or middle third of the tail (Table 4).

### 3.2. Correlation of the Studied Parameters

To investigate the correlation between tail length (‘TL’), tail circumference (‘TC’), vertebral number (‘nvertebrae’), and tail vertebral changes such as wedged vertebrae, block vertebrae, and axis deviation, these variables were correlated with one another. The results are presented in Table 5.

**Figure 5:**
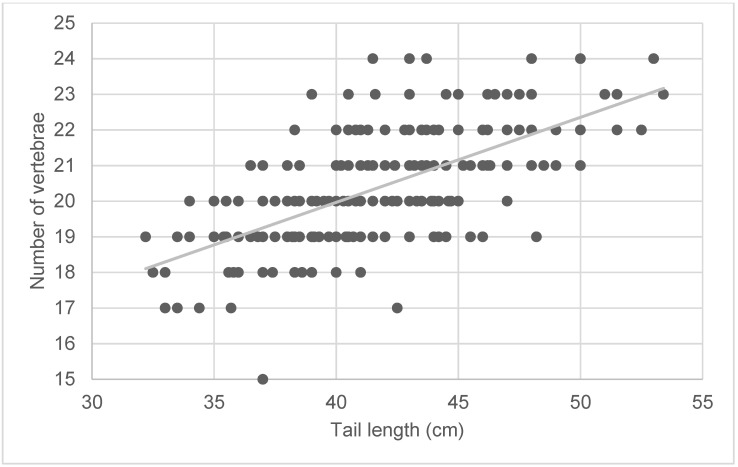
Symmetrical matrix representation for variables of tail length (cm) and number of vertebrae of lambs. The grey line represents the regression and was calculated as follows: y = 0.2387x + 10.419.

Pearson correlation coefficients yielded significant positive correlations between tail length and vertebral number (r = 0.63) (see also graphical representation in Figure 5), as well as tail length and tail circumference (r = 0.26; *p* <0.0001). Conversely, no significant correlation was found between tail circumference and vertebral number (r = −0.05).

As shown in Table 5, the incidence of fractures of the caudal spine was significantly correlated with tail length (r = 0.17) and vertebral number (r = 0.16), but not with tail circumference (r = 0.03).

The presence of wedged vertebrae was not significantly correlated with tail length (r = 0.03), tail circumference (r = −0.03), or number of vertebrae (r = 0.10), nor was the occurrence of block vertebrae (TL: r = −0.04, TC: r = 0.03, nvertebrae: r = 0.04). However, a significant correlation was demonstrated between the presence of wedged and block vertebrae (r = 0.15). Furthermore, axis deviations were significantly correlated with tail circumference (r = 0.19) and the occurrence of block vertebrae (r = 0.13). The other variables were not found to correlate with the occurrence of axis deviations.

The occurrence of block vertebrae did not seem to be influenced by the different effects tested (litter size, sex, number of previous lambings, ram, number of vertebrae) (Table 6). The parity and the number of vertebrae seemed to have a tendency for influence and the tail circumference showed a significant impact on the occurrence of wedged vertebrae. For the occurrence of fractures, a significant association with the tail length and the ram was found. The fact that tail circumference was significant in the axis deviation model with a *p*-value of 0.01 confirms the moderate correlation from the correlation analysis in Table 5 and suggests that animals with a larger tail circumference are more likely to exhibit axis deviations. Apart from this, a significant difference between female and male animals for the occurrence of axis deviations could be determined. The calculated *p*-values of the model effects are shown in Table 6.

The probability of the presence of wedged vertebrae, block vertebrae, and fractures in the tail area did not significantly differ between litter sizes (Table 7) and sex (Table 8). However, more fractures were observed when multiple lambs were birthed. The probability of the occurrence of tail axis deviations did not show any significant difference for litter size, but it did for sex (Table 7 and Table 8). Thus, with a probability of 11.8%, axis deviations occurred more frequently in female lambs than in male lambs (5.1%).

With regard to the occurrence of vertebral fractures, ram 1 differed significantly with 8.9% from the other three rams that were used, which had a probability of 22.9% and 33.6%. It was also shown that lambs with tails longer than 43 cm were up to twice as likely (31.7%) to have tail fractures as lambs with shorter tails (15.0–17.6%).

Lambs with a tail circumference greater than 14 cm were significantly more likely to have axis deviations than lambs with a smaller tail circumference (3.8–6.8%), showing a probability of 17.3%. In contrast, the occurrence of wedged vertebrae showed a significantly higher probability for lambs with an intermediate tail circumference (17.3%) than for lambs with thinner (4.7%) or thicker tails (3.5%).

## 4. Discussion

The study and morphological description of vertebral anomalies in the tail region, some of which result in visible tail abnormalities, represent an important step in the phenotyping of the caudal spine of sheep. Until now, such malformations could not be systematically recorded because tails were routinely docked, rendering assessment of tail abnormalities impossible. Due to increasingly stringent regulations of painful procedures on animals in Germany [16], breeding for short tails has become an important goal. In order to prevent the development of malformations in targeted breeding, the frequency and extent of vertebral anomalies in the tail region of sheep must be considered.

Using 2.667 lambs, the heritability mechanism of tail length was investigated in an Australian study. According to the results, tail length in Merino sheep had a heritability of 0.58 [17]. Oberpenning et al. also obtained a similar result with a direct heritability of 0.60 ± 0.08 for tail length. This high heritability for tail length suggests the possibility of producing a short-tailed sheep population within a few generations [18].

During a study on tail length in sheep, the so-called NoTail breed emerged, which exhibited a lethal factor called “sidewheeler”. Compression of the spinal cord nerves in the tail area led to increasing paralysis of the hindquarters in the tailless lambs during the rearing phase [19,20].

Several other studies have shown that the T gene is mainly responsible for tail length and influences the occurrence of vertebral deformities [21]. Mutations in the T gene cause early embryonic death in mice, altered vertebral shape in cats and cows [21,22], and increased embryonic mortality in dogs and sheep [23,24]. Radiographic examination of the caudal spine is suitable for visualizing any vertebral changes in sheep in the course of breeding for short-tailedness [15]. Such examinations can be performed under field conditions and are suitable for examining a large number of animals.

At present, there exists a dearth of literature investigating the radiological presentation of sheep tail structures, as well as studies documenting the occurrence of tail malformations. However, a study by Shelton [4] conducted radiographic and pathological examinations on three mouflons and Rambouillet sheep. Eleven caudal vertebrae were described in the mouflons and 18–24 in the Rambouillet sheep. Other authors described 3–24 tail vertebrae in sheep [25]. A radiographic study similar to our research was performed on the tail region of dogs [26], which also demonstrated tail malformations such as wedged and block vertebrae. The block and wedged vertebrae in the tail region of dogs were associated with vertebral anomalies in the cervical, thoracic, and lumbar spine. The authors of this study assumed that these types of vertebral malformations are anchored in a very early phase of embryonic development. When selecting suitable breeding animals, it should also be considered that the vertebral column and associated malformations develop from the same germ layer as the urinary tract [26]. These correlations could not be investigated in this study because no radiographic images of the cervical, thoracic, or lumbar spine of the animals were taken. This aspect should be considered in future research.

Since the lambs examined in the present study were not radiographically examined until 14 weeks of age, it was not possible to assess which abnormalities were congenital and which developed in the course of rearing. It can be assumed that fractures of the vertebrae arise from trauma, as per previous description of tread injuries in the tail area of undocked animals [1]. Whether these fractures result in ankyloses in the form of block vertebrae cannot be assessed at present due to the lack of radiographs during the development phase. As such, future imaging studies of lambs during the first weeks of life are necessary to further develop our understanding of the origin of lamb tail malformations.

In principle, block vertebrae are formed by the fusion of two adjacent vertebrae [27,28]. This fusion, which bridges the intervertebral gap, can lead to an axial deviation of the tail in the form of a kink [26,29]. However, other factors may also result in a kinked tail. In this study, for example, in addition to block vertebrae, associated separations in the intervertebral fissure and vertebral fractures were observed in connection with an axial deviation in the caudal spine. This complicates the phenotypic assessment of the caudal spine to the extent that no precise statement can be made about the presence of malformations without radiographic examination. This, in turn, may lead to axis deviations being incorrectly attributed to acquired malformations (such as vertebral fractures or cohesive separations), and a malformation being disregarded as the cause.

Another vertebral malformation that was frequently observed in this study was wedged vertebrae. This is a wedge-shaped bony vertebral structure that is inserted between two anatomically correct vertebrae [27,30]. It is not yet known to what extent vertebral anomalies (such as block and wedged vertebrae) affect the physiological function of the spinal cord. In order to assess the impairment of the spinal cord (for example, due to compression), magnetic resonance imaging (MRI) would be necessary, as X-rays only allow for one-dimensional imaging and are more suitable for showing bony structures.

This study demonstrated that the occurrence of block and wedged vertebrae correlate with each other and that block vertebrae in the tail area often occur in conjunction with axis deviations. Additionally, block and wedged vertebrae, as well as axis deviations, manifested primarily in the middle and/or caudal third of the tail. It was also found that the occurrence of block vertebrae does not seem to be significantly related to tail length, tail circumference, or vertebral number. This suggests that the development of such abnormalities is likely to be influenced by other factors, such as genetics. To investigate this, following generations of the animals with block vertebrae should be examined. In addition, even more animals should be bonitized to be able to collect a larger amount of data. In contrast, wedged vertebrae appeared to be significantly affected by tail circumference and showed a tendency to influence of the number of vertebrae. As our results showed a significant higher risk for lambs with an intermediate tail circumference, further studies on different sheep breeds should verify if this occurrence is breed specific.

Tail length and vertebral number seemed to be related to the development of vertebral fractures. As mentioned, it was shown that lambs with tails longer than 43 cm were twice as likely to have tail fractures as lambs with shorter tails. Therefore, it can be assumed that animals with longer tails are at increased risk of vertebral fractures. This represents, for the first time, a new argument for breeding for short tails and should not be neglected, especially regarding the importance of animal welfare and protection. A similar problem with long tails is known in hunting dogs. For many years, the tails of hunting dogs were docked due to injuries during hunting. However, a rethink is currently taking place there as well, since dogs need their tails for communication [31,32].

Thus far, the seminal reason cited for the intervention of docking lambs’ tails is to improve hygiene in the anogenital area. This is not only relevant for mating, lambing, slaughter, and shearing, but fundamentally for protection against the development of myiasis [1,33,34,35]. However, various studies have shown that the amputation of the caudal vertebrae in lambs causes significant pain reactions, which are indicated both by increased cortisol levels and by altered behaviour [12,13].

This study also showed that lambs with a larger tail circumference were significantly more likely to have axis deviations than lambs with a smaller tail circumference. Some studies have analysed the tail circumference of fat-tailed sheep and related it to the fat percentage of the carcass [36,37]. However, associations regarding tail abnormalities have not been examined thus far. In addition, female lambs were significantly more likely to exhibit axis deviations. This should be taken into account when selecting female offspring.

A zootechnical study of the correlation between tail length and body traits in Merinoland lambs indicated that lambs with long and woolly tails have an increased risk of contamination with dirt and dust. This is associated with impaired body weight development via increased susceptibility to infection. Based on this, the authors assumed a lower susceptibility to disease for short-tailed Merinoland bred ewes [18]. For this study 2.803 tail lengths records, 13.042 body weight records, 1.556 weaning weight records, and 3.986 post weaning weight records were analysed, which were documented over a 27-year period.

Most vertebral fractures are found in the caudal tail region, suggesting that the caudal region of the tail bones is more vulnerable to traumatic insults. In line with the goal of promoting animal welfare by not docking lambs’ tails, the occurrence of tail abnormalities and vertebral fractures should be critically assessed. It can be assumed that any vertebral fracture, tissue crushing, and/or contextual separation in the intervertebral region will result in pain, suffering, and damage. Often, these injuries remain largely unnoticed and are only discovered, for example, during tail shearing. Therefore, close animal observation is essential when keeping undocked sheep, not only when counteracting the development of a fly maggot infestation.

## 5. Conclusions

In order to better understand the development of deformities in the caudal spine in sheep and their clinical relevance, further investigations should be carried out at different developmental phases. In addition, acquired axial deviations, which are caused by vertebral fractures (among other things), should be considered a potential cause of pain. Furthermore, this study showed that females and animals with an increased tail circumference predominantly exhibited axis deviations. This should be considered when selecting potential breeding animals. Additionally, the proven ram effect on the occurrence of vertebral abnormalities should be considered.

## Figures and Tables

**Table 1:** Fracture frequency and location within the caudal spine of lambs.

Fracture Localisation in Thirds of the Caudal Spine	Absolute Frequency	Percentage of Total Population (%)	Percentage of Animals with Fractures (%)
No fracture	165	76.39	0.00
Caudal third	51	23.61	86.40
Middle third	6	2.78	10.20
Cranial third	0	0.00	0.00
Caudal and middle third	2	0.93	3.40

**Table 2:** Frequency and localisation of block vertebrae within the caudal spine of lambs.

Localisation of Block Vertebrae in Thirds of the Caudal Spine	Absolute Frequency	Percentage of Total Population (%)	Percentage of Animals with Block Vertebrae (%)
No block vertebrae	188	87.04	0.00
Caudal third	14	6.41	50.00
Middle third	13	6.02	46.43
Cranial third	0	0.00	0.00
Caudal and middle third	1	0.46	3.57

**Table 3:** Frequency and localisation of wedged vertebrae within the caudal spine of lambs.

Localisation of Wedged Vertebrae in Thirds of the Caudal Spine	Absolute Frequency	Percentage of Total Population (%)	Percentage of Animals with Wedged Vertebrae (%)
No wedged vertebrae	198	91.67	0.00
Caudal third	18	8.33	100.00
Middle third	0	0.00	0.00
Cranial third	0	0.00	0.00
Caudal and middle third	0	0.00	0.00

**Table 4:** Frequency and localisation of axial deviations within the caudal spine of lambs.

Localisation of Axial Deviation in Thirds of the Caudal Spine	Absolute Frequency	Percentage of Total Population (%)	Percentage of Animals with Axal Deviation (%)
No axis deviation	183	84.72	0.00
Caudal third	24	11.11	72.73
Middle third	9	4.17	27.27
Cranial third	0	0.00	0.00
Caudal and middle third	0	0.00	0.00

**Table 5:** Pearson correlation of tail length (‘TL’), tail circumference (‘TC’), number of vertebrae (‘nvertebrae’), occurrence of fractures, block and wedged vertebrae, and axis deviations in lambs. The Pearson correlation coefficient, the *p*-value, and the number of observations are reported.

	TC	TL	Fracture	Wedged Vertebrae	Block Vertebrae	Axis Deviation
nvertebrae	−0.05 0.47 216	0.63 <0.0001 216	0.16 0.02 216	0.10 0.14 216	0.04 0.52 216	0.09 0.17 216
TC		0.26 <0.0001 216	0.03 0.62 216	−0.03 0.62 216	0.02 0.72 216	0.19 0.00 216
TL			0.17 0.01 216	0.03 0.68 216	−0.04 0.60 216	0.04 0.53 216
Fracture				0.07 0.28 256	−0.04 0.49 256	0.07 0.24 256
Wedged vertebrae					0.15 0.02 256	0.08 0.20 256
Block vertebrae						0.13 0.03 256

**Table 6:** *p*-values of the global F-test for the model effects on the probability of occurrence of the four features wedged vertebrae, block vertebrae, fracture, and axis deviation.

Effect	Wedged Vertebrae	Block Vertebrae	Fracture	Axis Deviation
Litter size	0.97	0.23	0.38	0.93
Sex	0.52	0.85	0.74	0.04
Parity	0.06	0.58	0.58	0.14
Ram	0.85	0.44	0.06	0.29
TCclass ^1^	0.03	-	-	0.01
nVclass ^2^	0.09	0.19	-	-
TLclass ^3^	-	-	0.06	-

^1^ Tail circumference categories (<13 cm, 13–14 cm, >14 cm). ^2^ Number of vertebrae categories (<19, 19–20, >20). ^3^ Tail length categories (<38 cm, 38–42 cm, >42 cm).

**Table 7:** LSMeans for the probability of occurrence of wedged vertebrae, block vertebrae, fractures, and axial deviations in the tail region of lambs by litter size. Significant differences (*p* < 0.05) are marked with different superscript letters.

Litter Size	Wedged Vertebrae	Block Vertebrae	Fractures	Axial Deviation
Single	5.6% ^a^	25.5% ^a^	14.3% ^a^	6.7% ^a^
Twin	6.9% ^a^	11.6% ^a^	25.2% ^a^	7.5% ^a^
Triplet	7.9% ^a^	14.8% ^a^	23.8% ^a^	9.2% ^a^

**Table 8:** LSMeans for the probability of occurrence of wedged vertebrae, block vertebrae, fractures, and axial deviations in the tail region of lambs by sex. Significant differences (*p* < 0.05) are marked with different superscript letters.

Sex	Wedged Vertebrae	Block Vertebrae	Fractures	Axial Deviation
Male	8.0% ^a^	17.0% ^a^	21.5% ^a^	5.1% ^a^
Female	5.7% ^a^	16.0% ^a^	19.7% ^a^	11.8% ^b^

## Data Availability

The data presented in this study are openly available in JLUpub at doi: http://dx.doi.org/10.22029/jlupub-15650.
